# Insights into molecular and cellular functions of the Golgi calcium/manganese-proton antiporter TMEM165

**DOI:** 10.1016/j.jbc.2024.107567

**Published:** 2024-07-11

**Authors:** Stanislovas S. Jankauskas, Fahimeh Varzideh, Urna Kansakar, Ghaith Al Tibi, Esther Densu Agyapong, Jessica Gambardella, Gaetano Santulli

**Affiliations:** 1 Department of Medicine, Wilf Family Cardiovascular Research Institute, Einstein-Mount Sinai Diabetes Research Center (*ES-DRC*), Albert Einstein College of Medicine, New York City, New York, USA; 2 Department of Advanced Biomedical Sciences, “*Federico II*” University, Naples, Italy; 3 International Translational Research and Medical Education (*ITME*) Consortium, Academic Research Unit, Naples, Italy; 4 Department of Molecular Pharmacology, Einstein Institute for Aging Research, Fleischer Institute for Diabetes and Metabolism (*FIDAM*), Albert Einstein College of Medicine, New York City, New York, USA

**Keywords:** CDG, glycosylation, Golgi, lysosomes, Mn^2+^, SERCA, SPCA1, TMEM

## Abstract

The Golgi compartment performs a number of crucial roles in the cell. However, the exact molecular mechanisms underlying these actions are not fully defined. Pathogenic mutations in genes encoding Golgi proteins may serve as an important source for expanding our knowledge. For instance, mutations in the gene encoding Transmembrane protein 165 (TMEM165) were discovered as a cause of a new type of congenital disorder of glycosylation (CDG). Comprehensive studies of TMEM165 in different model systems, including mammals, yeast, and fish uncovered the new realm of Mn^2+^ homeostasis regulation. TMEM165 was shown to act as a Ca^2+^/Mn^2+^:H^+^ antiporter in the medial- and trans-Golgi network, pumping the metal ions into the Golgi lumen and protons outside. Disruption of TMEM165 antiporter activity results in defects in N- and O-glycosylation of proteins and glycosylation of lipids. Impaired glycosylation of TMEM165-CDG arises from a lack of Mn^2+^ within the Golgi. Nevertheless, Mn^2+^ insufficiency in the Golgi is compensated by the activity of the ATPase SERCA2. TMEM165 turnover has also been found to be regulated by Mn^2+^ cytosolic concentration. Besides causing CDG, recent investigations have demonstrated the functional involvement of TMEM165 in several other pathologies including cancer and mental health disorders. This systematic review summarizes the available information on TMEM165 molecular structure, cellular function, and its roles in health and disease.

The Golgi apparatus, also known as the Golgi complex, Golgi body, or simply Golgi, is a cellular organelle present in almost all eukaryotic cells. Despite being one of the first organelles identified in the cell, more than 120 years ago (1, 2), the Golgi remains one of the less studied organelles. The most investigated role of Golgi is its involvement in the glycosylation of proteins and lipids, primarily because disruptions in these processes lead to congenital disorders of glycosylation (CDG) (3, 4, 5). However, the Golgi is responsible for various other tasks in the cell, including protein sorting, regulation of endosome/lysosome homeostasis, ATG7-independent macroautophagy, storage of metal ions (with Ca^2+^ being the most significant one), and compartmentalization of phospholipase signaling (6, 7, 8, 9, 10, 11, 12, 13, 14).

The exact regulation of Golgi functions has long been unclear, but in the past decade, attention has turned to a new protein that plays a pivotal role in Golgi homeostasis, namely, Transmembrane protein 165 (TMEM165). TMEM165 was initially identified as a new causative factor of CDG, but further research has revealed its unique role in regulating Ca^2+^ and Mn^2+^ homeostasis, pH regulation, and overall Golgi function. Additionally, its involvement in several diseases beyond CDGs has been proved, further highlighting the understudied role of the Golgi in cellular physiology and pathophysiology.

## Evolution and structure of TMEM165

### Structural insights

Transmembrane protein 165 (TMEM165) is a protein encoded by the human gene *Tmem165*, which belongs to the Ca^2+^:H^+^ Antiporter-2 (CaCA2) family (2.A.106. according to the Transporter Classification Database https://www.tcdb.org) (15). It was previously known as ‘Uncharacterized protein family 0016’ (UPF0016) (16, 17). In most eukaryotic organisms, only single members of this family are present per genome, whereas in plant species genomes multiple CaCA2 genes can be found (18, 19).

The CaCA2 family is part of the larger lysine exporter superfamily, which includes transporters specific for Ca^2+^, Mn^2+^, Ni/Co, Fe/Pb, amino acids, glycolipid peptides, tellurium, and transmembrane electron carriers (20). All these proteins share the same evolutionary history, originating from a duplication of an ancient ancestor gene that encoded 3 transmembrane domains (20). This feature is observed in all modern members of the CaCA2 family, which typically have 6 or 7 transmembrane domains and a total protein length between 200 and 350 amino acids (15). Six of these domains are highly conserved across different taxa. For example, comparisons of human, avian, fish, insect, and cyanobacterium orthologs of *Tmem165* exhibited more than 60% homology in these domains (16). Similar results were obtained from comparisons of human *Tmem165* with its orthologs in the nematode, yeast, cyanobacterium, and plants (*e.g.* Arabidopsis) (21). However, the seventh transmembrane domain varies considerably between species in terms of amino acid sequence and position (whether N-terminal or C-terminal) or may even be absent altogether. For instance, *Gcr1*-dependent translation factor 1 (Gdt1), the yeast ortholog of the human *Tmem165* gene, contains 6 transmembrane domains. The heterologous expression of the full-length human *Tmem165* gene, which encodes 7 transmembrane domains, did not restore viability in yeast lacking gdt1, whereas a truncated variant of the human *Tmem165* gene lacking the seventh transmembrane domain fully rescued the phenotype (21, 22).

### Consensus motif of TMEM165

**Figure 1:**
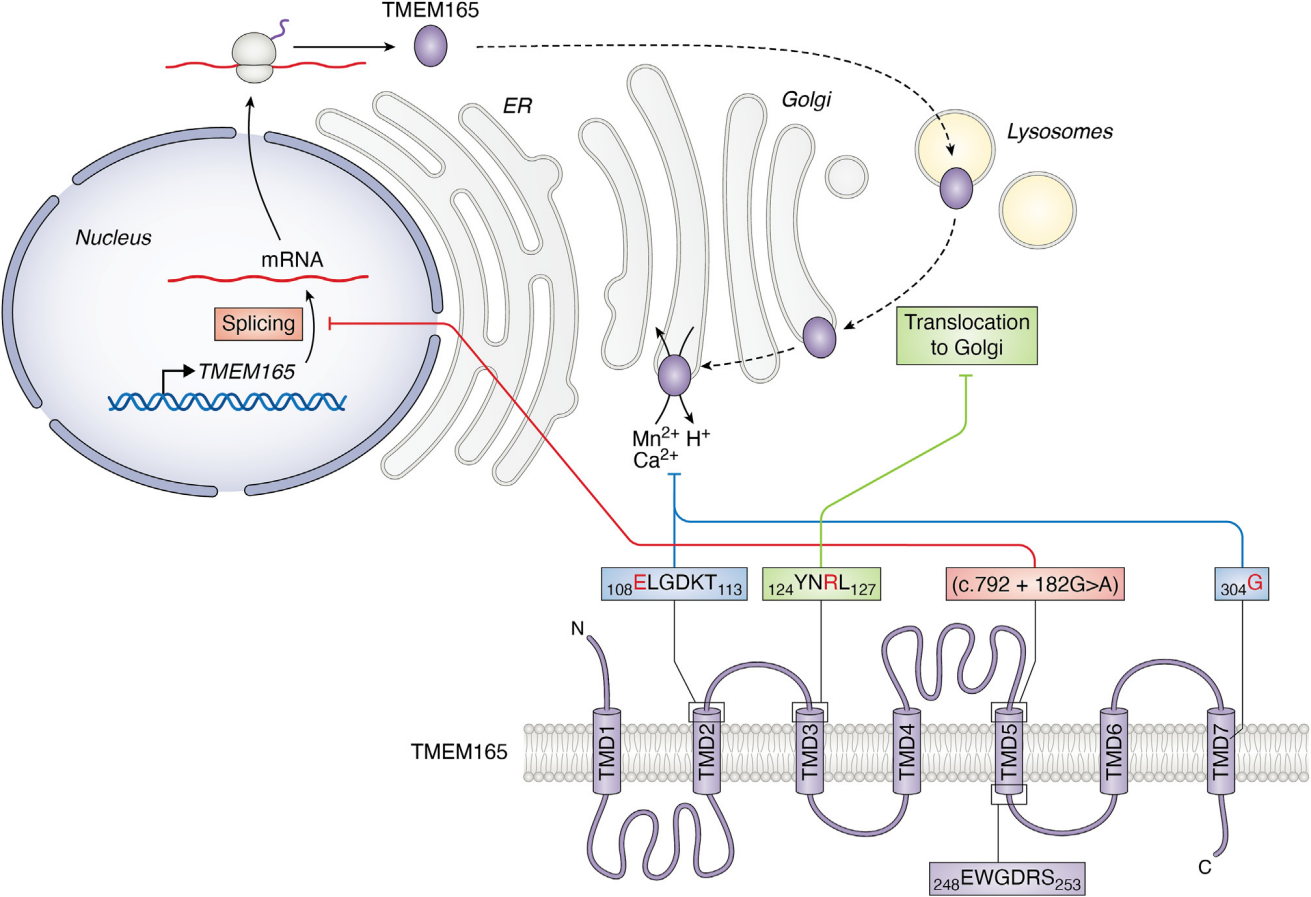
**Schematic representation of TMEM165-CDG causing mutations and proposed mechanisms of TMEM165 deficiency.** Human TMEM165 protein consists of 7 transmembrane domains (TMD). CaCA2 family signature consensus sequence E-φ-G-D-(KR)-(TS) is located in TMD2 and TMD5. This motif was demonstrated to be indispensable for the Ca^2+^/Mn^2+^:H^+^ antiporter activity of TMEM165. A lysosome targeting sequence YNRL is located at TMD3. Among patients with TMEM165-CDG 5 different disease-causing mutations were identified (marked in *red* on the figure). Point missense mutation [p.E108G] in TMD2 targets the CaCA2 family signature consensus motif disrupting the ability of TMEM165 to bind with Ca^2+^ and Mn^2+^. Two more-point missense mutations [p.R126H] and [p.R126C] are located in the lysosome targeting motif and plausibly hampers TMEM165 translocation to Golgi. Deletion mutation (c.792 + 182G > A) in TMD5 is causing the activation of a cryptic splice donor and the drastic decrease in mRNA expression of full-length TMEM165 protein. Finally, point missense mutation [p.G304R] in TMD7 indirectly affects the conformation of the Ca^2+^/Mn^2+^ binding site.

Another feature of CaCA2 proteins is the highly conserved consensus pattern E-φ-G-D-(KR)-(TS) (where φ can be any hydrophobic residue) (23). Due to a duplication event in the evolutionary history of the CaCA2 family, each protein contains two such motifs. Computational modeling of the 3D conformation of the human TMEM165 protein revealed that these consensus motifs are located in the midpoint of transmembrane domains 2 and 5 (Fig. 1), forming a loop that faces the cytosolic side of the membrane (15). These motifs have been shown to be necessary for two main functions of TMEM165 discovered so far, *i.e.* Ca^2+^/Mn^2+^ transport and maintaining the Golgi's ability to implement glycosylation (24, 25). The 3D modeling of TMEM165 suggests that both copies of the consensus motifs together form an ion binding site in the form of an "acidic cage" (15), similar to structures shown to bind divalent cations in other proteins (26, 27, 28). Cytosolic loops connecting the transmembrane domains of TMEM165 contain two lysosomal targeting motifs. These motifs are recognized by adaptor proteins AP1–4, which can recruit clathrin to initiate the formation of coated vesicles (29).

To date, no data are available on the transcriptional regulation of the *Tmem165* gene nor on TMEM165 post-transcriptional modifications.

### Alternative splicing variants of TMEM165

Evidence of an alternative splice variant of TMEM165 was found in human brain samples (30). One variant is 259 amino acids long and consists of 3 transmembrane domains, containing only one E-φ-G-D-(KR)-(TS) motif. The second splice variant is even shorter, with 129 amino acids and two domains, totally lacking the characteristic CaCA2 consensus motif (30). When overexpressed in HeLa cells, both splice variants show intracellular localization distinct from the canonical peptide. While the full-length TMEM165 resides in the Golgi (as discussed in detail below), both splice variants are localized in the endoplasmic reticulum (ER). The expression of these splicing isoforms does not rescue the defect in glycosylation of Lysosome-Associated Membrane Protein 2 (LAMP2) in TMEM165 knockout cells, which is traditionally used to measure the severity of glycosylation deficiency. However, they partially ameliorated the glycosylation of Trans-Golgi network integral membrane protein 2 (TGOLN2, also known as TGN46) (30). No more data are available on the splicing variants of TMEM165, and their abundance and functional role remain quite obscure.

## Molecular functions of TMEM165

### TMEM165 as Ca^2+^ transporter

Evolutionary and computational predictions of TMEM165's function as a Ca^2+^ transporter were proven in experiments conducted both in yeast and mammalian cells. *Saccharomyces cerevisiae (S. cerevisiae)* deficient for *Gdt1* (*Tmem165* ortholog) exhibited a decreased viability in high Ca^2+^ conditions (21, 31). The effects of *Gdt1* ablation were further exacerbated by the co-deficiency of the gene encoding plasma membrane ATPase-related 1 protein (pmr1) (yeast ortholog of the mammalian gene Ca^2+^-transporting ATPase type 2C member 1, *Atp2c1*) (21). *Atp2c1* (also known as secretory pathway Ca^2+^-ATPase pump type 1, SPCA1) and *Pmr1* encode a Golgi Ca^2+^/Mn^2+^ ATPase that pumps Ca^2+^ and Mn^2+^ into the Golgi lumen (32, 33). Thus, one may infer that both proteins play a similar role in Ca^2+^ clearance from the cytoplasm. Indeed, in yeast with double deficiency of *Gdt1* and *Pmr1*, Ca^2+^ concentration in the cytoplasm was shown to be higher than in yeast lacking *Pmr1* only (21). Additional confirmation came from experiments in which Gdt1 was expressed in the plasma membrane of the bacteria *Lactococcus lactis* (*L. lactis*). Direct measurement of intracellular Ca^2+^ demonstrated that GDT1 transports Ca^2+^ ions through the membrane (34). The heterologous expression of the human *TMEM165* gene in *S. cerevisiae* was able to rescue the phenotype of double *Gdt1/Pmr1* deficiency. Human TMEM165 protein expressed in *L. lactis* demonstrated the same ability to transport Ca^2+^ as GDT1 (22).

The ability of TMEM165 to clear Ca^2+^ from the cytoplasm has been also reported in mammalian cells; for instance, in HeLa cells overexpressing TMEM165, the increase in cytoplasmic Ca^2+^ upon treatment with thapsigargin (which releases Ca^2+^ from the ER) was lesser than in control cells (21). Targeted mutagenesis assays confirmed that the CaCA2 signature consensus motif E-φ-G-D-(KR)-(TS) plays an indispensable role in Ca^2+^ translocation through the membrane; in fact, the substitution of glutamate or aspartate in either of the two consensus motif sequences abrogates Ca^2+^ transport across the membrane (24).

### TMEM165 as Mn^2+^ transporter

Some members of the CaCA2 family proteins possess Mn^2+^ translocation activity, including the TMEM165 orthologs manganese exporter A (MneA) and manganese oxidase (Mnx) in bacteria, and photosynthesis-affected mutant 71 (PAM71) and chloroplast manganese transporter 1 (CMT1) in plants (35, 36, 37, 38). TMEM165 also appears to translocate Mn^2+^.

Yeast strains deficient for the Golgi Ca^2+^/Mn^2+^ ATPase *Pmr1* demonstrated reduced viability in high Mn^2+^ conditions, and Gdt1 co-deficiency exacerbated it (22, 39). Direct measurement of Mn^2+^ influx into *L. lactis* expressing GDT1 in the plasma membrane demonstrated the ability of the protein to translocate Mn^2+^ ions (39). However, the affinity of GDT1 for Ca^2+^ is 5 times higher than the one for Mn^2+^. The K_m_ for Mn^2+^ is estimated to be 83.2 ± 9.8 μM, whereas for Ca^2+^ is 15.6 ± 2.6 μM (39). The heterologous expression of the human *TMEM165* gene in *S. cerevisiae* was able to rescue high Mn^2+^-induced growth defect in yeast with co-deficiency of *Gdt1*/*Pmr1*, proving that human TMEM165 also possesses Mn^2+^ translocation activity (22). The heterologous expression of human TMEM165 in the membrane of *L. lactis* additionally confirmed this aspect (22). Importantly, targeted mutagenesis experiments demonstrated that the same amino acid in the consensus motif of the CaCA2 family was responsible for both Mn^2+^ and Ca^2+^ ion translocation (22).

The function of TMEM165 as a Mn^2+^ transporter was also documented in mammalian cells. In HeLa cells, *TMEM165* knockout did not affect Mn^2+^ levels in the cytoplasm but significantly decreased Mn^2+^ concentrations in organelles, further proving the role of TMEM165 as a Mn^2+^ Golgi transporter. TMEM165 knockout also retarded Mn^2+^ clearance from cells subjected to long exposure to high Mn^2+^ concentration (40).

Indirect evidence for TMEM165 Ca^2+^/Mn^2+^ transporter activity in the Golgi has been provided by *in vivo* assays. The majority of Ca^2+^ and Mn^2+^ content in milk derive from secretory vesicles dispatched from the Golgi compartment (41, 42, 43); the targeted deletion of *Tmem165* in murine mammary glands resulted in diminished concentrations of both Ca^2+^ and Mn^2+^ in milk with no changes in other divalent cations (44).

### TMEM165 as Ca^2+^/Mn^2+^:H^+^ antiporter

TMEM165-mediated Ca^2+^ and Mn^2+^ translocation activity was discovered to be coupled with the translocation of H^+^ in the opposite direction, making TMEM165 a Ca^2+^/Mn^2+^:H^+^ exchanger. This feature of TMEM165 was extensively studied by Pierre Morsomme’s group (45). They expressed *Gdt1* in the plasma membrane of *L. lactis* and measured pH, Ca^2+^, and Mn^2+^ concentrations. The rate of Ca^2+^ and Mn^2+^ translocation through the membrane appeared to be dependent on the pH level. The rate of cation transport increased together with the extracellular pH level. Conversely, the addition of extracellular Ca^2+^ or Mn^2+^ resulted in acidification of the extracellular media, showing that Ca^2+^ and Mn^2+^ translocation is inevitably coupled with H^+^ exchange (45). In yeast cells, deletion of *Gdt1* under normal conditions did not affect either cytoplasmic or Golgi pH levels, seemingly because of the robust function of vacuolar membrane ATPase. However, during glucose deprivation, the cytoplasmic pH of *S. cerevisiae* was lower in *Gdt1*-deficient cells (45). A realistic explanation is that upon ATP depletion, the direction of ion transport by TMEM165 is reversed to maintain a physiological pH within the Golgi. This mechanism is supported by similar data obtained in another independent study (31).

## Role of TMEM165 in cell function

### Tissue specificity of TMEM165

According to the Human Protein Atlas project, the *Tmem165* gene demonstrates ubiquitous expression throughout the organism with very low tissue specificity (46, 47). TMEM165 mRNA levels are relatively low, and on the protein level, no tissue specificity has been found for *TMEM165* either. Among 30 different tissues tested by single-cell RNA sequencing by the Human Protein Atlas (48), a specificity for *TMEM165* was only detected in the brain, where it was highly expressed in two clusters related to oligodendrocytes. In the other 29 tissues, *TMEM165* showed no specificity for any cell type.

### Cell compartmentalization of TMEM165

Several studies have shown that at the cellular level, TMEM165 localizes to the Golgi. The Golgi apparatus represents a series of flat membrane-enclosed disks known as cisternae, building up a stack. A stack is broken down into cis-, medial-, and the trans-Golgi network in which different steps of protein glycosylation are carried out (49). Testing the colocalization of *Gdt1* with different Golgi enzymes revealed that the protein resides in the cis- and medial-Golgi, but not in the trans-Golgi (21). In human fibroblasts, TMEM165 colocalized with both markers of cis- and trans-Golgi (16). However, upon dispersion of the Golgi structure with the help of the microtubule polymerization inhibitor nocodazole, TMEM165 maintained colocalization only with beta-1,4-galactosyltransferase, a trans-Golgi enzyme (16). This finding could be explained by the better resolution of Golgi compartments after nocodazole treatment, or it may indicate that TMEM165's Golgi retention mechanism is based on its retrograde transport, possibly explaining the Golgi localization of TMEM165 despite the absence of the ER-targeting sequence KDEL/HDEL (50, 51). Several lines of evidence support the idea of a retrograde transport of TMEM165 through the Golgi network. TMEM165 contains the lysosomal-targeting sequence YNRL (belonging to the classical YXXØ lysosomal-targeting signal) (52). *Au fait,* TMEM165 displayed colocalization with lysosomes/late endosomes and the plasma membrane, although to a lesser extent than with the Golgi (52, 53, 54). Finally, several TMEM165-CDG-causing mutations have been proven to decrease TMEM165 accumulation in Golgi but increase TMEM165 abundance in lysosomes, inferring the disruption of lysosome-to-Golgi transport of TMEM165 (52).

### Glycosylation defects caused by TMEM165-CDG

TMEM165 is necessary for accomplishing glycosylation in the Golgi. Mutations in the *TMEM165* gene cause type II CDG. At the cellular level, TMEM165-CDG is characterized by defective N-glycosylation; a relative increase in undersialylated and undergalactosylated glycans has been reported, pointing to a defect in both sialylation and galactosylation (16, 55, 56). Sialylation is performed in the trans-Golgi and most often is attached to galactose, while galactosylation precedes sialylation and occurs in the medial-Golgi (57, 58). Taken together, these observations suggest that TMEM165 deficiency most likely impairs enzyme(s) of the medial- and trans-Golgi. TMEM165-CDG has also been associated with more abundant fucosylated and high-mannose type N-glycans, denoting a decrease in N-glycan maturation (59).

Defective O-glycosylation has been detected in patients with TMEM165-CDG: It is characterized by a decrease in the monosialo- and an increase in the asialo-forms of Apolipoprotein C-III (16). Another evidence of defective O-glycosylation is the shifted ratio between nT-antigen (Galβ1-3GalNAc-α-Ser/Thr) and ST-antigen (NeuAcα2-3Galβ1-3GalNAc-α-Ser/Thr) (55).

TMEM165 deficiency also affects the glycosylation of lipids. Whereas normal cells show complex patterns of different gangliosides, TMEM165 knockout cells lack almost all of them, containing only trace amounts of GM2 and GM3 (60).

### TMEM165 mutations causing glycosylation defects

Mutations of the *TMEM165* gene provide important insight into TMEM165 cell functions (Fig. 1). Hitherto, 6 patients with TMEM165-CDG were described bearing 5 different mutations. One mutation (c.792 + 182G > A) causes the activation of a cryptic splice donor and the production of both full-size and truncated proteins (16). However, mRNA expression of full-length TMEM165 is drastically decreased in patients with this mutation, resulting in almost undetectable levels of mature protein (16, 52), attesting that the presence of TMEM165 itself is essential for a normal Golgi function.

The other 4 mutations provide more clues on the connection between the molecular function of TMEM165 and Golgi function. All these alterations represent point missense mutations resulting in a substitution of amino acid residues: i) [p.Arg126His] ii) [p.Arg126Cys] iii) [p.Gly304Arg] iv) [p.Glu108Gly] (16, 61). These mutations result in lower TMEM165 protein content compared to healthy controls (16, 52). However, the severity of glycosylation defects in cells from patients with a moderate decrease in TMEM165 abundance does not seem to be significantly different from patients with a virtual loss of TMEM165 protein (16).

Of these point mutations, two (i, ii) affect the lysosome targeting motif, one (iii) is located in the transmembrane domain of TMEM165 and does not target the motif with an established functional role, and one (iv) targets the signature consensus motif of CaCA2 proteins. Mutations affecting the lysosome targeting motif result in the retention of TMEM165 in the lysosome. Upon expression of the normal *TMEM165* gene in HeLa cells, approximately 50% of the protein colocalizes with lysosomes and 50% with the trans-Golgi. Expression of [p.Arg126His] and [p.Arg126Cys] TMEM165 shifts this ratio to 70 to 80% of TMEM165 located in the lysosomes and the rest in the trans-Golgi (52). Thus, functionally, the point mutations [p.Arg126His] and [p.Arg126Cys] exert similar effects as (c.792 + 182G > A): the latter deprives the Golgi of TMEM165 by a global decrease in its abundance, whereas the first two achieve the same goal by hampering protein transport. These data also show that the TMEM165-CDG phenotype is caused by TMEM165 deficiency disrupting Golgi function, and not other compartments.

The pathogenic mechanism of point mutations [p.Glu108Gly] and [p.Gly304Arg] is related to the direct disruption of TMEM165 molecular function and not to its abundance or localization. The expression of [p.Gly304Arg] *TMEM165* in HeLa cells did not result in its retention in lysosomes; on the contrary, it even increased TMEM165 targeting to the trans-Golgi (52). Mutation [p.Glu108Gly] directly affects the consensus motif 108E-φ-G-D-(KR)-(TS)113, substituting the glutamic acid (E108) thereby disrupting the structure of the "acidic cage" ion binding site (15). Computational prediction of TMEM165 conformations demonstrated that the [p.Gly304Arg] mutation also affects the structure of the "acidic cage" formed by the 108E-φ-G-D-(KR)-(TS)113 motif. When glycine is located in position 304, it does not interact with the consensus motif. However, the long side chain of arginine spans between the transmembrane domains 2 and 7 and interferes with the conformation of the 108E-φ-G-D-(KR)-(TS)113 motif. Modeling studies predicted that histidine or lysine in position 304 would not affect the "acidic cage" structure; indeed, the expression of TMEM165 with [p.Gly304His] or [p.Gly304Lys] rescued glycosylation deficiency in *TMEM165* knockout cells (15).

### Role of Mn^2+^ homeostasis in glycosylation defects caused by TMEM165 deficiency

One of the most plausible mechanisms by which TMEM165 deficiency causes glycosylation defects is a scarcity of Mn^2+^ content within the Golgi. This hypothesis is corroborated by several layers of evidence.

The first insight comes from the fact that mutations targeting CaCA2 family consensus motif and disrupting “acidic cage” recognizing Mn^2+^ and Ca^2+^ ions cause glycosylation defects (15, 62). At the same time, these mutations do not preclude TMEM165 localization to the Golgi (52). Thus, the ability to implement Ca^2+^ or/and Mn^2+^ transport is indispensable for the function of glycosylation enzymes in the Golgi.

The second and most compelling evidence is the rescue of glycosylation deficiency caused by either a pathogenic mutation in *TMEM165* or *TMEM165* knockout by Mn^2+^ supplementation (34, 40, 53, 60, 63). Moreover, the effect is dose-dependent: higher concentrations of supplemented Mn^2+^ are associated with lower electrophoretic motility of glycosylated proteins (56, 63). MnSO_4_ is as effective in rescuing glycosylation in TMEM165 knockout cells as MnCl_2_, which was used in other studies (60). Importantly, Ca^2+^ supplementation in *Gdt1*/*Pmr1* double-deficient yeast did not rescue the glycosylation effects (56). Disruption of the “acidic cage” structure impairs the ability of TMEM165 to transfer Ca^2+^ ions through the membrane as well as Mn^2+^ ions (22, 24); however, failure to rescue the glycosylation defect with Ca^2+^ supplementation strongly suggests that TMEM165-CDG is related to disturbed Mn^2+^ Golgi homeostasis.

The third argument in support of this hypothesis is that mutations in solute-carrier 39 A 8 (*SLC39A8*) are also known as a causative factor of CDG (64, 65). This gene encodes a plasma membrane transporter for bivalent ions, including Mn^2+^ (29, 66). Its deficiency reduces Mn^2+^ import into the cell, and may also result in depletion of Golgi Mn^2+^ content (67, 68). Finally, a number of enzymes involved in glycosylation use Mn^2+^ as a cofactor, mechanistically explaining why a decrease in Golgi Mn^2+^ disrupts glycosylation (69).

The knockout of another Golgi Mn^2+^ transporter, namely *ATP2C1* (encoding SPCA1) did not abolish the effect of Mn^2+^ supplementation (70). Inhibition of endocytosis also had no effect, showing that retrograde flow of Mn^2+^ is not able to overcome the Mn^2+^ deficit caused by TMEM165 knockout (70). Surprisingly, the capacity of Mn^2+^ to rescue TMEM165 knockout-induced glycosylation defects has been shown to be dependent on sarcoplasmic/endoplasmic reticulum Ca^2+^ ATPase 2 (SERCA2, encoded by *ATP2A2* gene). Its inhibition *via* thapsigargin totally abolishes Mn^2+^ effect, and ATP2A2 overexpression partially replicates the effect of Mn^2+^ supplementation (53, 70). These data imply that the rescue of TMEM165 knockout defect in glycosylation requires anterograde entrance of Mn^2+^ into Golgi, which could be mediated by SERCA2b; this pump possesses Mn^2+^ translocation activity but has much less affinity to Mn^2+^ compared to Ca^2+^ (71, 72, 73). An obstacle to this mechanistic explanation could be the fact that high Mn^2+^ concentrations inhibit SERCA2b (72). Nonetheless, the inhibition was observed with a concentration of Mn^2+^ that was ∼10 to 100 times higher than the concentrations of Mn^2+^ required for the rescue of the TMEM165 knockout phenotype.

### Role of pH in glycosylation defects caused by TMEM165 deficiency

A competing explanation of the pathophysiology of TMEM165-CDG implies the disruption of Golgi pH due to TMEM165 deficiency. TMEM165 acts as a Ca^2+^/Mn^2+^:H^+^ antiporter, regulating both cell and Golgi pH (45); pH levels are decisive for determining the correct conformation of proteins, affecting the function of enzyme active centers and the availability of glycosylation sites in proteins that require modification. Additionally, different compartments of the Golgi have different acidity levels, which are needed for specific functions (74, 75, 76, 77). Mutations in genes encoding subunits of V-ATPase, the main protein regulating Golgi pH, are known to cause severe CDGs (78, 79, 80, 81). However, despite the possibility of alterations in Golgi pH in TMEM165-CDG, the ability of Mn^2+^ supplementation to rescue glycosylation defects in TMEM165 knockout cells (40, 53, 60, 63) makes the "Mn^2+^ hypothesis" more favorable than the "pH hypothesis".

### Galactose supplementation rescues glycosylation defects caused by TMEM165 deficiency

The elusive aspect of TMEM165-CDG phenotype may relate to the ability of D-galactose supplementation to rescue glycosylation defects (53, 60, 63). Other monosaccharides were found to be ineffective (53, 60). Importantly, unlike the Mn^2+^ treatment, D-galactose failed to rescue O-glycosylation and restore ganglioside levels, being effective only in restoring the normal pattern of N-glycosylation (53, 60). Even in N-glycosylation, galactose supplementation demonstrated a lower efficacy compared to Mn^2+^. This aspect was evident in both a lower ratio of under-glycosylated to fully-glycosylated forms of proteins and a longer time required to achieve a beneficial effect *in vitro* (53, 60). Remarkably, the combination of galactose and Mn^2+^ supplementations has a synergistic effect. Furthermore, the combination of galactose and Mn^2+^ allows overcoming the inhibitory effect of thapsigargin, denoting how the galactose-mediated rescue of N-glycosylation is dependent on Mn^2+^ but not on the entry of these cations *via* SERCA2b (53). D-galactose has been previously proposed as an effective therapeutic strategy in CDGs of different etiologies, rescuing glycosylation defects caused by both Mn^2+^ deficit (SLC39A8-CDG) and uridine diphosphate (UDP)-Galactose deficiency (PGM1-CDG and SLC35A2-CDG) (82, 83, 84). The fact that many galactosyltransferases require Mn^2+^ as a cofactor makes the beneficial effect of galactose supplementation in TMEM165-CDG and SLC39A8-CDG even more enthralling (67, 69, 85, 86).

### TMEM165 degradation by Mn^2+^

**Figure 2:**
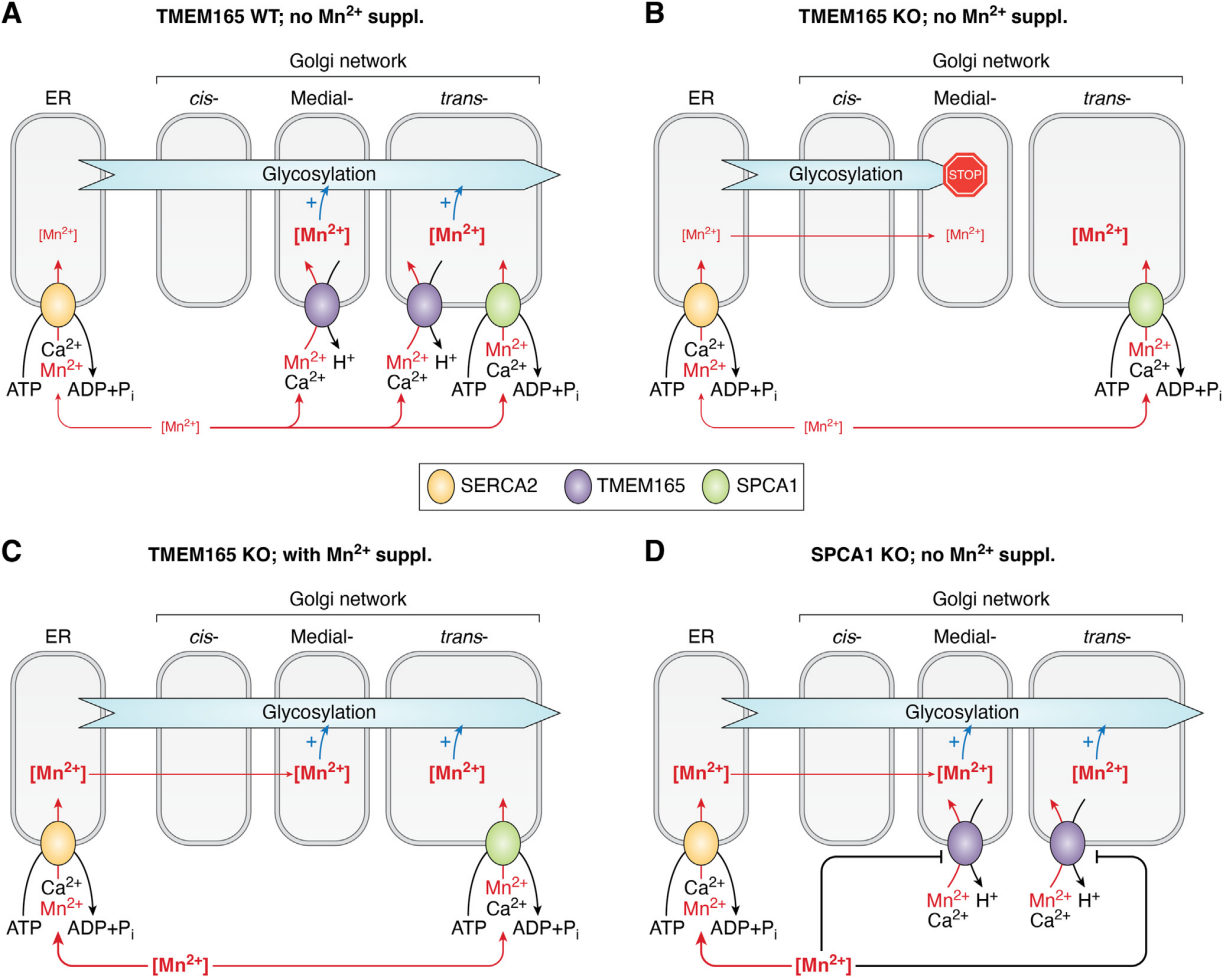
**Model of Golgi-ER regulation of Mn**^**2+**^**homeostasis.***A*, Under normal conditions, medial-Golgi is predominantly supplied with Mn^2+^ by TMEM165 and *trans*-Golgi by TMEM165 and SPCA1. Mn^2+^ import by SERCA2 is negligible. *B*, in the case of TMEM165 deficiency, medial-Golgi is deprived of Mn^2+^ halting the function of Mn^2+^-dependent glycosylation enzymes. This event is attributable to the low abundance of SPCA1 in medial-Golgi and the absence of retrograde Mn^2+^ flow from trans-Golgi to medial-Golgi. Most likely, the Mn^2+^ content in trans-Golgi is also decreased due to the absence of TMEM165 activity. Despite its ability to transport Mn^2+^, SERCA2 is not able to rescue the medial-Golgi Mn^2+^ insufficiency due to the low affinity to Mn^2+^; the high affinity of SPCA1 for Mn^2+^ eventually results in pumping all available Mn^2+^ into the trans-Golgi. *C*, with excessive Mn^2+^ supplementation, the SPCA1 Mn^2+^ translocation activity becomes saturated, giving the opportunity to SERCA2 to start pumping Mn^2+^ into the ER and potentially into the cis-Golgi. The anterograde flow of Mn^2+^ through the Golgi network supplies essential cofactors to the glycosylation enzymes and hence rescues the TMEM165-deficiency phenotype. *D*, SPCA1 deficiency results in massive Mn^2+^ accumulation in the cytosol. This phenomenon is firstly due to the loss of Mn^2+^ translocation into the Golgi lumen by SPCA1 *per se*. The increased concentration of cytoplasmic Mn^2+^ triggers TMEM165 degradation; thus, SPCA1 deficiency further augments the Mn^2+^ cytoplasmic level. A high concentration of Mn^2+^ allows SERCA2 to pump Mn^2+^ into the ER and subsequentially the Golgi, thereby preventing the disruption of the glycosylation processes.

The intimate connection between TMEM165 and Mn^2+^ involves not only the regulation of Mn^2+^ but also the control of TMEM165 levels by Mn^2+^ (Fig. 2). When cells are exposed to high Mn^2+^ levels, TMEM165 abundance decreases both in the whole cell lysate and in the Golgi compartment (62). Interestingly, the Mn^2+^ treatment does not seem to affect the expression levels of other Golgi proteins, showing a specific effect on TMEM165 (56, 62). Mn^2+^ supplementation causes TMEM165 to move to the lysosomes for degradation (62), such translocation to lysosomes and degradation of TMEM165 is observed when cells are treated with lysosome inhibitors, such as chloroquine and leupeptin (62).

The rate of TMEM165 clearance strictly depends on Mn^2+^ concentration, with higher Mn^2+^ concentrations leading to faster degradation (62). No other divalent cations, including Mg^2+^, Ca^2+^, Cu^2+^, Zn^2+^, Co^2+^, had the same effect, indicating the specificity of TMEM165 for Mn^2+^ (62). Various studies suggest that the degradation of TMEM165 requires specific recognition of Mn^2+^ by the CaCA2 family consensus motif E-φ-G-D-(KR)-(TS). Skin fibroblasts from TMEM165-CDG patients harboring the [p.Glu108Gly] mutation exhibited a modest TMEM165 clearance under high Mn^2+^ conditions; in contrast, cells from patients with [p.Arg126His/Cys] mutations displayed TMEM165 degradation, though still slower than healthy donor cells (62). As mentioned earlier, replacing Glu108 disrupts the "acidic cage" of the E-φ-G-D-(KR)-(TS) motif, which is critical for binding Mn^2+^ and Ca^2+^. On the other hand, substituting Arg126 reduces the retention of TMEM165 in the Golgi. Yet, when cells are treated with high concentrations of both Mn^2+^ and Ca^2+^, the degradation of TMEM165 is significantly slowed down (62). These observations also suggest that TMEM165 translocation from Golgi to lysosomes requires binding of Mn^2+^ to the CaCA2 signature motif, as this motif is also responsible for binding Ca^2+^ ions. Since TMEM165 has a higher affinity for Ca^2+^, the inhibitory effect of Ca^2+^ on Mn^2+^-induced degradation of the protein may be attributable to competition for the same active site.

Experimental data strongly suggest that TMEM165 degradation is triggered by Mn^2+^ binding to the protein from the cytoplasmic side, as deduced from the fact that *ATP2C1* knockout or loss-of-function mutations in *ATP2C1* induce TMEM165 degradation (40, 62, 87, 88). SPCA1 encoded by the *ATP2C1* gene imports Mn^2+^ into the Golgi and its deficiency robustly augments Mn^2+^ concentration in the cytosol (40). Moreover, upon SPACA1 deficiency, downregulation of TMEM165 is also caused by its translocation to lysosomes, as in high Mn^2+^ treatment (87, 88). Additional support for this hypothesis may come from the fact that the overexpression of *ATP2A2* partially rescues TMEM165 expression in SPCA1-deficient cells (87). If SERCA2b re-supplies the Golgi with Mn^2+^, then its overexpression should decrease cytoplasmic Mn^2+^ concentration in the absence of SPCA1.

Interestingly, the rescue of glycosylation deficit caused by TMEM165 ablation is achieved already at a concentration of 1 μM of MnCl_2_ (56), whereas a robust downregulation is observed at 25 μM (62). One might infer that a potential biological role of Mn^2+^-induced TMEM165 degradation is to preclude Golgi overload with Mn^2+^. However, in pathological conditions like Hailey-Hailey disease, caused by dysfunction of SPCA1, this mechanism becomes detrimental (32, 88).

### Signaling effects of TMEM165-deficiency

TMEM165 deficiency has been shown to affect signaling pathways mediated by Transforming growth factor beta (TGFβ) and Bone morphogenetic protein (BMP) (89). In the chondrogenic cell line ATDC5, *Tmem165* deletion results in lower levels of phosphorylation of Mothers against decapentaplegic homolog (SMAD) 2 and higher levels of phosphorylation of SMAD1,5,9; similar alterations have been detected in fibroblasts isolated from patients with TMEM165-CDG (89). SMAD2 and SMAD1,5,9 are key mediators of TGFβ and BMP signaling, respectively (90). The expression of *Tgfβ* is drastically reduced in *Tmem165*^*−/−*^ cells; the expression of the TGFβ target gene *Serpine1* is also reduced, proving the loss of TGFβ signaling (89). In addition to reduced *Tgfβ* expression, *Tmem165*^*−/−*^ cells also have reduced levels of TGFR2 and higher expression of Asporin, which blocks the interaction of TGFβ with its receptors. However, the downregulation of *Tgfβ* expression is most likely to be the main cause of TGFβ signaling loss, as *Tmem165*^−/−^ cells are still able to respond to exogenous TGFβ stimulation (89).

*Tmem165*^−/−^ cells display an increased expression of BMP and two of its receptors (namely BMPR1b and BMPR2); in addition, the expression of a negative regulator of BMP signaling – Noggin – is decreased (89). Treatment of control cells with MnCl_2_ has the same effects as *Tmem165* ablation, *i.e.* stimulation of BMP signaling and downregulation of TGFβ signaling (89).

It is important to note that the loss of Ca^2+^ pumping activity due to TMEM165 deficiency most likely induces Ca^2+^-mediated signaling. The data on these phenomena are scarce; however, an increased phosphorylation of Calcium/calmodulin-dependent protein kinase type II (CaMKII) has been reported in *Tmem165*^−/−^ cells (89).

### Role of TMEM165 in lysosomal function

Intriguingly, in a recent study conducted on *Caenorhabditis elegans*, Zajac and collaborators identified a conserved gene, *lci-1*, which represents the worm counterpart of the human *TMEM165,* showing its involvement in enabling lysosomal Ca^2+^ entry in a pH-dependent manner (54). Through two-ion mapping and electrophysiology, TMEM165 was shown to function as a proton-activated lysosomal Ca^2+^ importer (54). These data were substantiated by the measurement of lysosome pH in fibroblasts from healthy donors and patients harboring mutations in *TMEM165*. The majority of the fibroblast batches, isolated from different TMEM165-CDG patients, had a lower lysosomal pH alongside an increased number of lysosomes (28). Moreover, silencing *TMEM165* expression in HeLa cells resulted in acidification of the lysosomal compartment (28).

These findings are captivating, especially considering that, as discussed above, TMEM165 abundance in lysosomes is relatively low compared to its levels within the Golgi. Since defects in lysosomal Ca^2+^ channels are linked to several neurodegenerative diseases (91), understanding lysosomal Ca^2+^ importers could offer new insights into the physiology of Ca^2+^ channels.

## Role of TMEM165 in health and disease

### TMEM165 and lactation

TMEM165 plays a fundamental role in lactation by regulating the levels of Mn^2+^ and Ca^2+^ in milk. Its expression peaks during lactation (*Tmem165* expression was found to be ∼25 times greater during peak lactation compared to early pregnancy) and declines rapidly after the lactation period ends (92). In mice lacking TMEM165, milk composition is altered, with lower levels of lactose and higher levels of calcium, iron, zinc, fat, and total protein (44). The effects of the quality of the milk were also observed in the pups of the lactating mice, where pups nursed by Tmem165 knockout mothers had significantly lower weights. Since TMEM165 acts as a Ca^2+^/Mn^2+^:H^+^ antiporter, its deficiency results in the accumulation of H^+^ ions, reducing lactose synthesis and osmosis-mediated dilution of milk. This study emphasizes the importance of TMEM165 in milk production and provides insights into its cellular functions.

### Clinical manifestations of TMEM165-CDG

Glycosylation plays a vital role in the functioning and stability of proteins in various cellular processes. Defects in genes responsible for glycosylation lead to many pathological manifestations. Mutations in several genes cause CDGs, with mutations in the Phosphomannomutase 2 (*PMM2*) gene being the most prevalent (93). CDGs are classified as type I or type II, where type I is associated with a defect in the assembly or transfer to the peptide chain, and type II is associated with processing or remodeling. TMEM165 initially caught attention as a culprit in one of the CDGs (16, 52, 59). TMEM165-CDG falls into type II, accounting for approximately 8% of CDG-II patients, and is inherited in an autosomal recessive manner (16, 93).

Clinical manifestations of TMEM165-CDG vary across different systems in the body. Patients typically exhibit psychomotor and growth retardation, facial dysmorphism, skeletal anomalies, hepato-splenomegaly, muscular hypotrophy, feeding problems, and abnormal fat distributions (16, 59). Skeletal anomalies are prominent, possibly due to the role of glycosylation in extracellular matrix proteins and bone metabolism. TMEM165 deficiency may also affect chondrocyte maturation and hypertrophy, contributing to skeletal issues (59, 89). Other symptoms include strabismus, ptosis, white matter abnormalities, pituitary gland hypoplasia, fever episodes, transient epilepsy, cardiovascular defects, restrictive lung pathology, and renal failure (16, 59, 61).

Various therapeutic approaches for TMEM165-CDG have been explored. Mn^2+^ supplementation helps suppress glycosylation defects by restoring Golgi Mn^2+^ homeostasis (62). D-galactose supplementation has similar effects, improving glycan structures and patient well-being (60). Genetic therapies, including mutation-specific antisense therapy, hold promise for restoring normal TMEM165 protein levels (94).

### Toward *in vivo* models of TMEM165-CDG

In order to understand TMEM165-CDG consequences, researchers have developed animal models with manipulated TMEM165 expression. For instance, *Danio rerio* (zebrafish) has been utilized to study the effects of TMEM165 deficiency on skeletal abnormalities and biochemical disturbances (95). Early research in yeast demonstrated abnormalities in the glycosylation processes due to a deficiency in a yeast ortholog of TMEM165 (56). Zebrafish, harboring a TMEM165 protein that is 79% identical to the human one, has been explored as a potential model. Injection of antisense morpholinos into zebrafish embryos inhibited *Tmem165* expression. Similar to yeast, *Tmem165*-deficient zebrafish exhibited glycosylation defects with decreased N-linked glycan abundance (95). Additionally, morphant zebrafish displayed abnormal cartilage development with craniofacial defects and increased chondrocyte differentiation markers along with decreased osteoblast maturation factors (95). These findings mirrored results in mouse ATDC5 cells and human HEK293 cells, where *TMEM165* knockout induced early chondrogenic differentiation and mineralization, somehow elucidating the skeletal abnormalities (89).

### TMEM165 and cancer

In addition to CDGs, TMEM165 has been linked to other human diseases, particularly cancer. Overexpression of *TMEM165* has been observed in various types of cancers. For example, breast cancer cells show higher TMEM165 levels compared to normal cells, and increased TMEM165 expression correlates with worse outcomes in breast cancer patients (96). Similarly, in hepatocellular carcinoma, TMEM165 is overexpressed in cancer cells, promoting invasive activity (97). These observations suggest that TMEM165 could serve as a biomarker and therapeutic target for breast cancer and hepatocellular carcinoma.

In murine models, TMEM165 overexpression was associated with increased invasive activity in certain types of cancer (97). To further investigate these phenomena, mouse xenograft models were used to test the effects of TMEM165 knockout in breast cancer tumors (96). Similar to the changes in N-glycosylation observed in zebrafish, mice injected with *Tmem165* knockout cells exhibit reduced tumor growth and vascularization, alongside decreased vimentin and increased E-cadherin levels within the tumors. These findings indicate that TMEM165 may play a role in enhancing cancer invasion capabilities.

### TMEM165 and other human diseases

Single-nucleotide polymorphisms in the *Tmem165* gene have been associated with mood disorders. Specifically, a polymorphism in the *TMEM165* gene, rs534654, has been linked to an increased risk of bipolar disorder type 1 (98). This polymorphism is located near the Circadian Locomotor Output Cycles Protein Kaput (*Clock*) gene, which is a circadian gene. Circadian genes have previously been associated with an increased risk of mood disorders, which may explain the connection between this *Tmem165* polymorphism and bipolar disorder (98). On the other hand, there seems to be a negative correlation between *Tmem165* and autistic spectrum disorder (ASD): serum extracellular vesicles (EVs) obtained from ASD children aged 3.5 years were found to express TMEM165 mRNA in significantly lower levels compared to controls (99); several other genes involved in the glycosylation process were also downregulated in ASD (99). Decreased levels of TMEM165 seem to be part of a general glycosylation disturbance in these patients, rather than being the causative factor.

Most recently, the overexpression of TMEM165 protein was also detected in the saliva of COVID-19 patients with an active infection, along with other molecular alterations. This finding sheds light on potential mechanisms by which the disease can disrupt cellular processes and metabolism (100).

## Conclusion

Fine-tuning TMEM165 activity may contribute to maintaining cellular Ca^2+^ and Mn^2+^ homeostasis. While there have been some preclinical investigations on TMEM165 using experimental animal models, research in this area remains limited. Historically, studies of TMEM165 have focused on understanding TMEM165-CDG mechanisms and their role in glycosylation, primarily using *in vitro* approaches. However, recent findings on the involvement of TMEM165 in cancers and the association of specific polymorphisms with mental health disorders strongly suggest that alterations in TMEM165 expression could play significant, tissue-specific, roles in various pathologies. Another unexplored aspect is the post-translational modification of TMEM165.

## Data availability

All data generated in this study are provided in the article and its supplementary materials.

## Conflict of interest

The authors declare the following financial interests/personal relationships which may be considered as potential competing interests:

Dr Santulli is an Editorial Board Member for *JBC* and was not involved in the editorial review or the decision to publish this article. The other authors declare that they have no conflicts of interest with the contents of this article.
